# Re-do aortic operation in a young patient for aggressive Takayasu’s arteritis

**DOI:** 10.1186/1749-8090-7-91

**Published:** 2012-09-26

**Authors:** Ioannis Bougioukas, Dimitrios Mikroulis, Aron Frederik Popov, Georgios Bougioukas

**Affiliations:** 1 Department of Thoracic and Cardiovascular Surgery, University of Göttingen, Robert-Koch-Strasse 40, Göttingen, 37075, Germany; 2 Department of Cardio-Thoracic Surgery, University Hospital Alexandroupolis, Alexandroupolis, Greece

**Keywords:** Arteritis Takayasu, Aortic dissection, Bentall procedure

## Abstract

Takayasu’s arteritis is an inflammatory arteriopathy which involves the aorta and its major branches, causing mainly stenosis of their lumen, though aneurysmal lesions can also occur. A young female with Takayasu’s disease presented to our hospital with acute lung oedema due to severe aortic insufficiency and ascending aorta dilatation. She had already undergone distal ascending aorta and hemiarch replacement due to Standford type A aortic dissection five years ago. The patient had also undergone reconstruction of abdominal arteries’ stenoses with extraanatomical bypass. We performed a Bentall procedure with a valved conduit and implantation of the coronary ostia as buttons on the conduit. A mechanical valved graft was used instead of a bioprosthesis, due to possible early degradation of a bioprosthesis. The postoperative course was uneventful and the one year follow-up was normal. Valve-sparing aortic root replacement should be avoided in Takayasu’s arteritis due to high rate of recurrent regurgitation.

## Background

Takayasu’s arteritis is an inflammatory arteriopathy which involves the aorta and its major branches, causing mainly stenosis of their lumen, though aneurysmal lesions can also occur. We report a case of a young female with Takayasu’s disease presenting with ascending aorta dilatation and severe aortic insufficiency five years after distal ascending aorta replacement due to aortic dissection type A.

## Case presentation

A 24 year-old Caucasian female was admitted to our hospital with acute pulmonary oedema due to severe aortic valve insufficiency. The patient was already diagnosed for Takayasu’s disease five years ago, as she was subjected to replacement of the distal ascending aorta and hemiarch because of acute aortic dissection type A. Later she was operated for stenosis of celiac artery and superior mesenteric artery, and stenosis of the orifices of both renal arteries, implying a relative aggressive form of the disease. A graft was then used to direct blood flow from the right internal iliac artery to all stenosed arteries. This operation was complicated by acute thrombosis of right renal artery graft, leading to early urgent reoperation for right nephrectomy.

We operated on the patient soon after she recovered from the pulmonary oedema. She was on steroid treatment since the diagnosis of her disease. At the time of operation, the erythrocyte sedimentation rate (ESR) was 16 mm/h, and C - reactive protein (CRP) concentration was 6.3 mg/dl (normal < 0.5). The procedure was performed through a median sternotomy. After re-sternotomy, the left femoral artery and the right atrium were cannulated. The left ventricle was vented through the right superior pulmonary vein. After fibrillation of the heart, the artificial graft was clamped just proximal to the origin of the brachiocephalic artery. The residual aneurismal ascending aorta was incised and Calafiore cardioplegia was given directly into the coronary ostia while the operation was performed with moderate hypothermia. The aortic root was significantly dilated and the aortic wall was thickened. The tricuspid aortic valve was excised and a composite graft with a 23 mm mechanical valve (St. Jude Medical Masters HP Valved graft / St. Jude Medical, Minnesota, USA) was implanted. The coronary ostia were harvested as buttons and were re-implanted to the graft with a running 4–0 prolene suture. Finally, the distal anastomosis of the composite graft with the old graft was performed with a running prolene suture. Reinforcement of the anastomosis was not necessary. The patient’s postoperative course was uneventful and she was discharged from the hospital on the 10^th^ postoperative day. Part of the dilated aortic root wall was sent for histological examination, which verified the diagnosis of Takayasu’s arteritis. The one year clinical follow-up of the patient was remarkable with normal echocardiographical results and she was doing well in normal daily activities.

## Discussion

Takayasu’s arteritis is a chronic inflammatory arteriopathy that predominantly affects young women. Aortic insufficiency is considered an important risk factor for mortality in patients with this disease and its incidence is reported between 13 and 25% [1]. The main cause of aortic valve regurgitation is annular dilatation resulting from severe dilatation of the ascending aorta. Surgical treatment includes aortic valve replacement (AVR) or composite graft replacement (CGR). The fragility of the aortic wall and annular tissue can cause valve detachment after AVR or anastomotic aneurysm after CGR [2].

Matsuura et al. reported their study of 90 patients with Takayasu’s arteritis who underwent surgery for aortic valve regurgitation. Patients positive for CRP (>1.0 mg/dl) or whose ESR was higher than 20 mm/h were placed on steroid therapy until these values were normalized [3]. They performed aortic valve replacement in 63 patients and composite graft replacement in 27 patients. Detachment of the prosthetic valve occurred in 7 patients with AVR, and detachment of the proximal anastomosis of the prosthetic graft occurred in 1 patient with CGR. A bioprosthetic valve had been used in 2 patients with valvular detachment. Late dilatation of the residual ascending aorta occurred in 7 patients of the AVR group and in 1 patient of the CGR group. The authors performed valve-sparing aortic root replacement in 4 patients, 3 of whom (75%) subsequently required AVR for recurrent aortic regurgitation in the follow-up period. In their conclusions, Matsuura et al. mentioned that a straightforward comparison of the results of the AVR and the CGR groups was not possible, as the follow-up period for the AVR patients was longer than for the CGR patients and no significant statistical difference in late aortic events between the two groups was observed.

Furukawa et al. reported on a case of severe calcification of the bioprosthetic valve of a female patient who had undergone a Bentall procedure for aortic insufficiency and ascending aortic aneurysm [4]. They noticed severe calcification of the body and the base of the cusps of the bioprosthetic valve. The time interval between the first Bentall procedure and the re-operation was seven years.

Osamu et al. presented 17 cases (15 patients) with aortic regurgitation caused by aortitis [5]. Of these patients 11 were diagnosed as having Takayasu’s arteritis. They performed aortic valve replacement in 4 patients and aortic root replacement in the rest of them. Two patients with AVR needed a composite graft replacement due to aortic root aneurysm and prosthetic valve detachment, 12 and 7 years after the first operation, respectively.

Our patient had undergone replacement of the distal part of the ascending aorta and small part of the arch, because of acute dissection type A. Five years later she presented at our institute with acute pulmonary oedema related to aortic regurgitation due to aortic root dilatation. Her history of vascular surgery due to stenosis of celiac, superior mesenteric and renal arteries suggested an aggressive type of the disease. She was already on steroid treatment (prednisolone), whereas the blood inflammation values (CRP, ESR) implied an ongoing inflammation.

After reviewing the literature regarding of this disease, we excluded an aortic root remodeling considering the high risk of failure and the need of an additional risky cardiac operation. An ascending aorta replacement with a composite graft was decided.

Our patient was a young female with a child wish in the future. Due to the severity of her medical history, we felt that the use of a bioprosthesis instead of a mechanical valve would be a possible menace to her life given the high probability of an early second cardiac reoperation because of early calcification and dysfunction of the tissue valve. Nevertheless, we discussed with her thoroughly the therapeutical options that could be offered, and finally she decided for the implantation of a mechanical valve conduit.

## Conclusion

In conclusion, valve-sparing aortic root replacement should be avoided in Takayasu’s arteritis due to high rate of recurrent regurgitation. Composite graft replacement may offer a more secure long-term result in comparison with AVR, although no significant statistical difference between the two techniques has been observed. The implantation of mechanical or bioprosthetic valve should be based on patient’s choice and should be tailored for each patient individually in terms of advantages and disadvantages. Patients with serological values positive for active inflammation should be put – when possible - on steroid treatment before the procedure.

## Consent

Written informed consent was obtained from the patient for publication of this Case report and any accompanying images. A copy of the written consent is available for review by the Editor-in-Chief of this journal.

## Abbreviations

AVR: Aortic valve replacement; CGR: Composite graft replacement; ESR: Erythrocyte sedimentation rate; CRP: C-reactive protein.

## Competing interests

The authors declare that they have no competing interests.

## Authors' contribution

IB is the author, GB is the surgeon, DM made the literature review, AP participated in manuscript corrections and gave important comments. All authors read and approved the final manuscript.
